# Clinician’s use of automated reports of estimated glomerular filtration rate: A qualitative study

**DOI:** 10.1186/1471-2369-13-154

**Published:** 2012-11-23

**Authors:** David H Smith, Jennifer Schneider, Micah L Thorp, Suma Vupputuri, Jessica W Weiss, Eric S Johnson, Adrianne Feldstein, Amanda F Petrik, Xuihai Yang, Susan R Snyder

**Affiliations:** 1 The Center for Health Research, Kaiser Permanente Northwest, Portland, Oregon; 2 Department of Nephrology, Kaiser Permanente Northwest, Portland, Oregon; 3 The Center for Heath Research, Kaiser Permanente Southeast, Atlanta, Georgia; 4 Department of Nephrology, Oregon Health and Sciences University, Portland, Oregon; 5 Centers for Disease Control and Prevention, Atlanta, Georgia

**Keywords:** Estimated glomerular filtration rate, Qualitative, Serum creatinine

## Abstract

**Background:**

There is a growing awareness in primary care of the importance of identifying patients with chronic kidney disease (CKD) so that they can receive appropriate clinical care; one method that has been widely embraced is the use of automated reporting of estimated glomerular filtration rate (eGFR) by clinical laboratories. We undertook a qualitative study to examine how clinicians use eGFR in clinical decision making, patient communication issues, barriers to use of eGFR, and suggestions to improve the clinical usefulness of eGFR reports.

**Methods:**

Our study used qualitative methods with structured interviews among primary care clinicians including both physicians and allied health providers, recruited from Kaiser Permanente Northwest, a non-profit health maintenance organization.

**Results:**

We found that clinicians generally held favorable views toward eGFR reporting but did not use eGFR to replace serum creatinine in their clinical decision-making. Clinicians used eGFR as a tool to help identify CKD, educate patients about their kidney function and make treatment decisions. Barriers noted by several clinicians included a desire for greater education regarding care for patients with CKD and tools to facilitate discussion of eGFR findings with patients.

**Conclusions:**

The manner in which clinicians use eGFRs appears to be more complex than previously understood, and our study illustrates some of the efforts that might be usefully undertaken (e.g. specific clinician education) when encouraging further promulgation of eGFR reporting and usage.

## Background

Pre-dialysis chronic kidney disease (CKD) is a common condition with an estimated prevalence of more than 13% of the US population [1]. But less than half of patients with CKD are unaware they have it [2] or don’t carry a diagnosis in their medical record [3], suggesting low patient and provider awareness. Historically, serum creatinine has been the principal way of assessing kidney function. But serum creatinine is an imperfect method of identifying diminished kidney function. The use of a formula that includes serum creatinine age, sex, and race produces an estimate of the kidney’s glomerular filtration rate (eGFR) which improves the diagnostic accuracy over serum creatinine alone. Recently, automated reporting of patients’ eGFR by clinical laboratories has been promulgated as a method for increasing CKD awareness and, therefore, also improving appropriate treatment and follow-up. For example, automated reporting of eGFR by clinical laboratories has been encouraged to by the National Kidney Foundation [4] and National Kidney Disease Education Program [5] and is required by at least six states [6].

Using eGFR has its own disadvantages, such as underestimating renal function in healthy individuals, leading to increased false positive diagnoses [7]. Even so, a recent systematic review found that automated eGFR reporting was associated with changed care patterns, including increased nephrology referrals and consultations [8]. But as noted in that review it is unclear how eGFR reporting may have caused those changes.

Many factors likely influence how health care providers use laboratory tests. For example, a provider’s knowledge of the patient’s clinical situation, their reimbursement, and understanding of the sensitivity and specificity probably all influence how often a test is ordered and how it is interpreted and used clinically [9]. As a result, efforts to influence the laboratory ordering patterns of physicians have met with varying success [10,11]. Little is known about how clinicians (i.e. both physicians and allied health care providers) understand and respond to laboratory reports of renal function, or whether they respond to and interpret eGFR differently than serum creatinine. To help fill that gap we undertook a qualitative study using structured interviews to enhance our understanding of how primary care clinicians use eGFR in clinical decision making, patient communication issues, barriers to use of eGFR, and suggestions to improve the clinical usefulness of eGFR.

## Methods

### Study site and systems

The study was conducted in 2010 (April through June) at a nonprofit group-model health-maintenance organization (HMO), Kaiser Permanente Northwest (KPNW), in Washington and Oregon. The site has 15 medical centers and approximately 485,000 members. Electronic databases provided administrative and clinical data and a full electronic medical record (EMR) has been in place since 1996. The study was approved by the institutional review board at KPNW and clinician participants provided written informed consent.

The EMR and related systems used at KPNW contain several tools that clinicians can use to automate functions. Several of these automated tools were referred to by our participants so we give a brief description of them here. The Panel Support Tool (PST) is a ‘dashboard’ indicator of potential care gaps that are reported at the patient level. For example, it reports on the current status of testing and follow-up for patients with diabetes, cardiovascular disease, asthma and CKD, among other co-morbid conditions, and recommends treatment and testing strategies to close the care gaps. The EMR also allows the creation of ‘dot phrases’ that can be used, for example, to automatically populate text in clinical notes and in patient letters.

### Estimated GFR (eGFR) reporting by laboratory

Our study was designed to take advantage of a systematic change at KPNW where all laboratory locations began automatically reporting eGFR routinely with serum creatinine in its laboratory results to clinicians. This reporting began on February 1, 2004. Prior to this, only the serum creatinine value was reported to clinicians on laboratory reports. The laboratory used the 4 variable version of the Modification in Diet and Renal Disease (MDRD) Study [12], which includes a binary variable for whether the patient is black. Because race data were not available, the laboratory reported eGFR values for both black and non-black routinely with each serum creatinine result. eGFR values above 60 ml/min/1.73 m^2^ were reported at “>60”. No further comments or information was provided.

### Qualitative methods

Qualitative methods are effective strategies for documenting and analyzing unique, complex social phenomena, such as clinician experiences with a “newly” reported lab value [13-15]. Qualitative data, such as interviews, can reveal information unanticipated by researchers [16-18], and may be key to helping us understand how clinicians’ are interacting with automatic reporting of the eGFR. Additionally, individual interviews are designed to elicit the participant’s perspective and experience on a topic, and are therefore particularly useful in defining the range and variability of beliefs, behaviors, and experiences of study populations, as well as the natural language people use to discuss these issues [13,14,19]. To this end, primary care clinicians’ experiences of the automatic laboratory reporting of the eGFR were captured and analyzed through a series of 30 minute, structured, open-ended face to face interviews.

### Recruitment and study participants

We identified a list of 139 Family Practice (FP) or Internal Medicine (IM) based primary care providers (PCPs) who had been employed with KPNW from at least January 2002 to the present. This time frame was chosen so that clinicians could speak about their experiences of eGFR both before and after automatic reporting began in 2004. We included clinicians who were either physicians (MD) or allied health practitioners (NP or PA). We aimed to interview a minimum of 16 PCPs, a number we determined as sufficient for reaching redundancy of information and themes based on prior experience with qualitative methods and interviewing clinicians. Of these 16 clinicians, our goal was to interview 8 allied health practitioners (distributed equally between IM and FP), and 8 MDs (distributed equally between IM and FP). We also attempted to balance the participants geographically across the 13 clinics. Clinicians were recruited by email, sent by the Chief of Nephrology (co-author MLT), inviting them to participate in a 30 minute structured interview. Lunch was provided to the participants. We completed 19 in-depth individual interviews with PCPs. Of these, 13 were MDs (8 IM, 5 FP) and 6 were allied health practitioners (2 IM, 4 FP). We sent 89 individual recruitment emails to reach this total, with 64 participants providing no response to the email and 6 participants indicating scheduling conflicts or lack of time as their reason for not participating.

### Data collection and analysis

The research team developed a structured guide (based upon prior experience [16-18,20] and a literature review) which was refined following the first few interviews. With each participant we followed the interview guide, which consisted of 15 key questions and approximately 45 follow up prompts. The guide elicited information about clinicians’ overall reaction to the automatic reporting of the eGFR, barriers and facilitators to the use of the eGFR, work practices related to the use of the eGFR, and overall advice on how to improve it. Interviews were conducted by a trained, third-party qualitative methodologist not known to the participants [co-author JS], audio-recorded, and professionally transcribed for analysis. Analysis was led by co-author JS with guidance and input from the research team. Analyses focused on representing, describing, and interpreting data, using standard techniques [15,19,21,22] and a qualitative research software package, *ATLAS.ti* 5.0 (Scientific Software Development, 1997) to code data and generate reports of coded text for analysis. We developed a coding dictionary based on the interview guide and review of the transcribed interviews. Transcribed interviews were coded by marking passages of text with phrases indicating content of the discussions. Using the report and query functions of Atlas.ti, coded text was further reviewed through an iterative process, resulting in refined themes [22,23].

## Results

We interviewed 19 clinicians, 10 in the department of IM, 9 in the department of FP (Table 1). Thirteen of the 19 were physicians, 2 nurse practitioners and 4 physician assistants. The average number of years that the clinician participants worked at KPNW was 16.3 years for physicians and 18.5 years for allied health practitioners. The average panel sizes (i.e. number of patients assigned to the clinician for health care management) were 1251 for the physicians and 1176 for the allied health practitioners. Eight clinicians worked full time and 11 part time. While our interviews occurred approximately 6 years after the 2004 initiation of automatic reporting of eGFR, participants had no trouble recalling when the shift to automatic reporting began, nor any hesitation describing their reaction, feelings, or impact on workload both at the onset of the reporting and over time.

**Table 1:** Participant Demographics

	**PCP**	Allied*	**Totals**
	**(n = 13)**	**(n = 6)**	**(n = 19)**
**Provider Type**	8 IM; 5 FP	2 IM; 4 FP	10 IM; 9 FP
Yrs at KP**	Range 2–30;	Range 11–22;	
	Ave: 16.3	Ave: 18.5	
**Clinical Allocations**	4 FTE; 9 PTE	4 FTE; 2 PTE	
**Panel Size**	Range 700–2044;	Range 1000–1405;	
	Ave: 1251	Ave: 1176***	
Other Roles****	9	2	

* 2 nurse practitioners; 4 physician assistants;

** Includes all clinicians with date of service from at least January 2002 to present (gaps in employment permissible).

*** 2 Allied physicians without their own patient panel to manage.

**** Other roles include such things as: provider/resident education and communication; urgent care; clinical director; team lead; health plan board member; document and coding; recruitment and retention; and home health/hospice work.

### Clinician use of eGFR: before and after automated reporting

We asked clinicians about their use of eGFR prior to, and after, automated eGFR reporting was instituted. About half of the physicians said they had, at least sometimes, calculated eGFR before the implementation of automated reporting, while none of the allied health professionals did so. In fact, all the clinicians said they had primarily used serum creatinine as their gauge of kidney health before automated reporting (Table 2).

**Table 2:** Comparison of Use of eGFR Value Prior to, and After, Automatic Reporting (n = 19)

**Use of eGFR Value Before Automatic Reporting**				
**Common Theme and Key Findings**	**PCP**	**Allied**	**Totals**	**Illustrative Quotes**
	**(n = 13)**	**(n = 6)**	**(n = 19)**	
***Provider Calculated eGFR Prior***				*I would sometimes calculate it [eGFR]to see, but it wasn’t common. And that was generally if their creatinine wasn’t normal, just so I’d have a better idea of how bad their GFR was. But I wouldn’t if they were older and they had a normal creatinine*. – IM PCP
· sometimes to occasionally	6	0	6	
· rarely to never	7	6	13	*No, I never calculated it [GFR]… I would just look at their creatinines and if they were elevated…*. – FM NP
***Provider Primarily Utilized Creatinine***				
· yes	13	6	19	*… all I ever did [prior] was keep track of patients’ creatinines*. – FM PCP
**Use of eGFR Value After Automatic Reporting**				
**Common Theme and Key Findings**	**PCP**	**Allied**	**Totals**	**Illustrative Quotes**
	**(n = 13)**	**(n = 6)**	**(n = 19)**	
***Changed Overall Approach to CKD Management***				*There’s a number of folks [with Stage] 2 CKD …, I feel like it helps me manage them ****a lot ****better*. –FM NP
· yes	8	4	12	*Definitely it has shifted my clinical practice…I think I run less and less into trouble ordering certain medications.* – IM PCP
· no / not much	5	2	7	*It hasn’t necessarily changed my approach… It may have just labeled them as having now another problem*. – IM PCP
***Utilization of eGFR & Creatinine Values in Decision-making***				
· uses both	11	4	15	*Now I look at the trends in both creatinine and their GFR. And I do update in the patient’s problem list…* - IM PCP
· uses eGFR more often	2	1	3	*I do look at the trend of GFR, and that helps me, first of all, in updating the diagnosis or putting the diagnosis in the problem list… It’s very helpful. So, yeah, I do look [eGFR] up a lot.* – IM PCP
· uses creatinine more often	0	1	1	

When asked about whether their overall approach to CKD management had changed since automated eGFR reporting, more than half of the clinicians said that it had. However, a minority said their overall approach had not changed, and by clinician type, there seemed to be little difference in whether management of CKD had changed. We found that the majority of clinicians reported currently using both eGFR and serum creatinine in clinical decision-making. Only one clinician reported currently using serum creatinine more often than eGFR.

### Benefits and challenges of automated eGFR

Clinicians’ perceived benefits of automated eGFR included time savings, increased disease awareness and improved patient care. Clinicians mentioned that having the eGFR calculated saved them valuable clinic time because it streamlined their work and removed the need for calculating it themselves. For example an internal medicine clinician commented (Table 3):

[Previously] I would very frequently have to look up their kidney function and actually calculate the GFR…So, definitely in an older population you encounter that often with certain medications. When automatic reporting came on, it was really helpful. I’ve never calculated it since then, and I said to myself, ‘Oh, that’s so going to save me time!’.

**Table 3:** Overall Impact of eGFR Automatic Reporting: Benefits and Challenges (n = 19)

**Benefits**	
**Common Theme and Key Findings**	**Illustrative Quotes**
***Time Savings***	*I thought it was great [to have it automatically reported], because I didn’t have to try to manually calculate it. Prior I had been using kind of just ballpark numbers to try to guesstimate when I thought somebody’s renal function was starting to decline and if I needed to adjust medication. So, it was challenging because it added work to my day to have to manually do that or try to assess that… So it has made life easier for me to have it calculated*. – FM PCP
· automatic calculation and reporting makes approach and work to CKD management more streamlined	
· easier to have eGFR calculated for provider - saves valuable clinic time to not calculate equation on own when they need it	
	*I think it’s a good tool. So the fewer steps that we have to do to get to the right answer, and the right thing to do, the better it is. I think the automatic calculator is quicker and better at math than I am, and more reliable. And so, it takes away some of the potential for error that I might have introduced by manually doing the calculations myself.* – IM PCP
***Grateful for the Information***	*Well, what it did was show that there were a lot of people with worse renal function than we had appreciated previously based just on creatinine. … we started looking at treatment of Stage 3′s, …trying to put [them] on ACE inhibitors. It wasn’t a usual practice until that [automatic reporting] happened. And without [automatic reporting], it would be very difficult to do*. – IM PCP
· providers wish they had the automatic eGFR value prior	
· providers feel the missed opportunity to help some patients by not having the automatic value previously	
· believe it to be a good clinical tool	*The ongoing reaction I’ve had [to the automatic reporting], is wishing I had known this a longtime ago. … It just makes me think of all the stuff I wasn’t doing or keeping track of before. -* FM NP
· helpful to have a more precise picture of renal health and CKD staging than just creatinine could provide	
	*I find it is really helpful. I think it’s a useful tool to use as a screen. And I really think it’s an important item to have on the problem list so that you pay attention when you’re prescribing… it becomes part of your decision making if you’re going to moderate dosing of medication.* – IM PCP
***Increased Awareness***	*It helps me when I review the charts for certain treatments or just to get to know the patient. It makes me aware that I can’t order certain medications. And it definitely makes me aware that I should check it at regular intervals to make sure I don’t miss when it starts trending down.* – IM PCP
· created more awareness of and attention to tracking CKD in general	
· now know about and can manage all the patients provider did not know about before automatic reporting began that have a “normal or slightly abnormal” creatinine and an abnormal eGFR	*Well, it’s made more awareness of chronic kidney disease. It’s sort of opened up a whole new [population] of chronic kidney disease patients. And it’s probably a more sensitive number to follow, rather than the creatinines…I can have a creatinine of 1.4 in one person, with a normal GFR, and a creatinine of .9 with an abnormal GFR - the GFR is simpler to pull. So ultimately I like it better*. - FM PCP
· identified a pool of patients on providers’ panel with CKD status much worse than the creatinine value alone was indicating – would not have “known” about these patients or referred on to Nephrology without automatic reporting	
	*Some of the patients who had a mildly abnormal creatinine I’m now finding have a much more reduced GFR, and that puts them in a higher stage of chronic kidney disease. And I am picking up a few of those that I wouldn’t have known before and then referring them on…It’s just that it alerts me to the fact that their kidney disease is worse than I might have suspected just from the level of creatinine.* – IM PCP
***Improve Patient Care and Management***	*It’s very much helped with the care of patients. I feel like I know what’s going on all the time now and I do a better job…I feel like all the people I didn’t, you know, do right by before - and that kind of kills me - I feel like I give them good care finally.* –FM NP
· overall provide better patient care by having the automatic eGFR value	
· improves the ability of providers to assess and act on a patient’s renal health and functioning earlier or to determine appropriate referral to Nephrology at earlier time points	*If there’s a low GFR but their creatinine is normal, I might have ignored that before…[because] I thought [their renal function] was normal. So that’s the major difference …because before if the creatinine was one, I might not have ordered it for another year. Whereas now, if it reflects a low GFR I might order it more often, or start adjusting the medicines. So I’m definitely ordering more tests.* - FM PCP
· greatly helps in medication management efforts, including determining both the appropriate type and dosage of medication · having the more sensitive value of the eGFR, along with creatinine, helps give gradation and refinement to patients’ renal health – helps provider determine “just how bad” a patient may or may not be	*And the one thing that I feel it does do, if a patient goes into a hospital, having GFR value [CKD stage] on the problem list will protect him from somebody starting a dangerous medication and monitor it more closely… I want to optimize the person’s blood pressure. I want to optimize their sugar control. I want to be careful with what medications I’m using… monitoring them with the GFR has more meaning than monitoring them with the creatinine. So it’s nice to have it more available now.* - IM PCP
· helps provider manage the Medicare refresh diagnosis process related to CKD status	
**Challenges**	
**Common Theme and Key Findings**	**Illustrative Quotes**
***Patient Confusion / Fear***	*Some patients were shocked – they were dismayed. They wanted to talk to me. I had some people who just couldn’t understand, asking ‘What’s wrong with my kidneys?’… FM NP*
· initially caused some otherwise healthy patients concern and upset regarding “suddenly” having a CKD diagnosis	
	*So, I think it caused distress and some fear because I never told them anything was wrong before, or I was waiting until their creatinine got high before I figured it out*. - FM PCP
· initially caused some otherwise healthy patients undue fear and stress regarding their kidney health and future possibility of dialysis	*It’s still a bit of an issue where there is that disconnect between some of the older patients who have a normal creatinine but their GFR is in the chronic kidney disease range. So it generated for some patients’ questions, concern, alarm that I think wasn’t really necessary*. – IM PCP
***Increase Provider Workload***	*So basically, there might have been fifty or a hundred people who I had considered normal, who all of a sudden had CKD3, by the GFR definition. So really, it’s more work, but if that’s the definition, then that’s the definition, and it’s real. So I’ve made mechanisms to deal with it.* – FM PCP
· initial reporting created a “new”, “unknown”, and “larger” pool of patients in Stage 3 that now needed outreach and follow-up	
· initially created a “thinking” burden when trying to determine the correct e GFR value on lab report – (both African American and Caucasian values reported)	*I think the automatic reporting is an absolutely great idea. But, the actuality is we’ve got some problems with how the computer reports it…I should not have to read every time the African and non-African American values when the computer could somehow designate that. If we have race in our computer, it should be automatically reported as the [correct] GFR and the computer should be estimating for the race. I shouldn’t have to be wasting one second of time thinking about that…It hasn’t created clinical harm, but it’s just extra thought and extra work for me*. – IM PCP
· generated more follow up and tracking work for providers – another condition to now follow and manage	
· extra time and workload for provider to create their own systems and processes for tracking, monitoring, and following up on patients eGFR values and renal health	
	*Initially I felt there was a little confusion to patients around giving the limits of normal GFR, so that the patient who was older could have a normal creatinine but an abnormal GFR, and the lab result would indicate chronic kidney disease. And it just added a little extra problem of something you either had to explain to the patient, or that they would ask questions about…I would usually then end up having to generate some sort of a letter explaining it, rather than just sending out a copy of the lab report, which made more work for me*. – IM PCP
· extra time and workload for provider to address patient fears and concerns regarding meaning of eGFR value and CKD stage/status (phone calls, creating patient letters)	

Clinicians also discussed being appreciative of the information, and wished they’d had the information earlier because there were patients in whom opportunities for clinical intervention were previously missed. They said eGFR, and the subsequent staging of CKD, gives them a better picture of renal health than they could get with serum creatinine alone. Clinicians mentioned that their awareness of CKD was greater with eGFR being automatically reported. For example, before automated reporting some patients with a normal serum creatinine were missed as having CKD. They said like the reporting allowed them to identify those patients and take appropriate action like referral to nephrology. While it is recommended at KPNW that patients be referred to a nephrologist when their eGFR falls below 30, there are no barriers to referral at any level of kidney function. Clinicians reported improved patient management because it allowed them to assess and act on patient’s renal health at earlier stages than with serum creatinine alone. Additionally they noted that the appropriateness of medication and medication dosing was improved. They also discussed organizational financial improvements related to more accurate diagnosis, specifically Medicare.

Several concerns of eGFR reporting were also noted, including patient confusion and increased clinician workload. At KPNW it is common for patients to be sent a record of their laboratory values, including automated eGFR reporting. Especially in the initial phase of automated reporting, some patients ‘suddenly’ had kidney dysfunction, causing patient confusion and some anxiety over their health. For example, patients were confused about the new information including seeing two values of eGFR (one for black, and one for non-black), and were also concerned about their risk of renal dialysis. Addressing these patient concerns translated into added workload for clinicians by necessitating phone calls and explanatory letters to be sent. The dual reporting of two values by race also caused a ‘thinking’ burden for clinicians since they were not able to simply examine the eGFR value without also determining the patient’s race. Perhaps the most important burden perceived to clinician workload was that of adding another disease to manage, because eGFR reporting revealed a new and potentially quite large group of patients to manage. Allied Health providers reported being less likely to incorporate a diagnosis of CKD into the patient’s health record, and some clinicians reported creating systems to monitor and track eGFR values for their patients.

### Changes in work practices related to CKD management

Table 4 illustrates work practices findings influenced by eGFR automated reporting, stratified by MD and allied health clinicians. Approximately half (53%) of the clinicians reported they increased the amount of patient education they provided following eGFR automated reporting. Allied health practitioners and family practice MDs were more likely to report this increase in patient counseling and education than internal medicine MDs. Additionally, 68% of the clinicians noted the onset of eGFR automated reporting created a need for designing and implementing additional follow-up communication strategies in the form of specialized letters and telephone ‘talking points’ for explaining eGFR results to their patients.

**Table 4:** Comparison of Work Practices Related to CKD Management since Automatic eGFR Reporting (n = 19)

**Common Theme and Key Findings**	**PCP**	**Allied**	**Totals**	**Illustrative Quotes**
	**(n = 13)**	**(n = 6)**	**(n = 19)**	
***Patient Communication and Activation Activities***				*If it is under 60, I tell the patient about what’s going on. I spend time trying to educate the patient on that and things to avoid to stay as healthy as possible…I do talk to and counsel people more now than I ever have before, on the NSAID usage or abuse. And it may correlate in terms of timing with that [automatic reporting]. – IM PA*
· increased counseling / education discussions with patients about GFR value, kidney health, and CKD management	6 (yes)	4 (yes)	10	
	7 (no)	2 (no)	9	
				*I use the eGFR information quite a bit… have a conversation when people need more information about protecting their kidneys if they get down to a GFR below 60. So, if their cholesterol is high, give them information about their cholesterol. If their GFR is low, try to get them to do some things to protect their kidneys, etc. – FP PA*
				*I am more aggressive about counseling for prevention of kidney disease, now… I start talking with them about making sure they’re getting plenty of water, avoiding caffeine, making sure their blood pressure is in control, making sure they’re not on high protein diets that are more challenging for the kidney to filter, etc. … and so just a lot of a more aggressive lifestyle counseling for things that may maintain or maybe even improve kidney function. – FP PCP*
				*It depends on the patient and what I’m explaining, whether or not I go into it with them. Obviously, if they ask me, then I tell them. Generally I don’t bring it up – it’s more how I practice what I’m doing. – IM PA*
· created specialized letters and phone talking points for explaining eGFR results and follow up activities to patients	9 (yes)	4 (yes)	13	*I send the patient a letter, using a dot phrase which described the patient’s kidney function – explains that if it’s a little bit off, it could be for many reasons, and that often it is age and kidneys, but that at this point it’s not concerning and we want to re-check in 3 months. – FP NP*
	4 (no)	2 (no)	6	
				*It does generate the need for additional information. I have dot phrases that I use to explain GFR, just to let them know the filtration rate and what that means. I have dot phrases that explain where they can get more information from the [National Kidney Foundation]– FP PCP*
				*So I have devised my own method that seems rational to me…I have a dot phrase for when the creatinine is normal, and the GFR is low and it says, ‘Your creatinine is normal but your GFR is low, sometimes this can be due to diabetes or high blood pressure, and a lot of times in three months it changes back to normal, so let’s check it in 3 months…’ etc. – IM PCP*
***Overall Referral Patterns to Nephrology***				*Yes, I am possibly referring more [to Nephrology]…I would have to say about 5 percent more, and chart review is my first request. – IM PA*
· subtle increase (approx. 1 to 2 month)	8	6	14	
				*It’s probably a subtle increase, because the information is so clearly in your face, as a clinician you can just see it. It would be hard to ignore, or hard to get distracted and not pick it up, the way it’s reported. – NP IM*
				*I think it has increased the number…probably once a month I’m referring someone - maybe once or twice a month - to the nephrologist…for a face to face or chart review, it varies. –FP PCP*
				*It has some because… once it gets down towards thirty, then I’ll refer them to Nephrology to take a look at it. And sometimes if I’m really not sure and it’s getting closer and I’m worried about something or other, I’ll have the nephrologist do a chart review. – IM PCP*
· no perceived increase in referrals	4	0	4	*It’s not had much impact on my referral to nephrology because usually I don’t refer unless it gets much lower than that. So for the mildly decreased GFR’s of 45 to 60 range, I’m still managing it. – FP PCP*
				*No, I don’t refer more… I just treat them. They only ones that get a chart review are the Stage Fours. And they’re not that many of those. – IM PCP*
· believe referrals have decreased	1	0	1	*I’ve probably cut down the number of referrals to Nephrology… I don’t know that I referred a lot before the change, but I would say in general probably it’s reduced the amount of referrals because I can monitor more, and try to help prevent them from progressing. I can do interventions earlier. – FP PCP*
***Typical GFR Referral “Cut-off” Values***				*Well, it’s probably when it gets down closer to 40 or below I’m more likely to do a referral to Nephrology and specify a chart review. …If it’s hovering around 60, or if it’s in the mid-fifties, I don’t feel so compelled to do anything about it, right then, except to make sure that the patient gets rechecked again in 6 months, or something. – IM NP*
· eGFR value low 40’s to 40	1	4	5	
				*I believe it’s 40 or 45, is the cutoff point between seeing the Nephrologist or not… if it’s in that 40 range or so, then usually I go ahead and refer at some point. Usually I follow them for at least a few months and have done the other lab work and ultrasound within that timeframe. – FP PCP*
· eGFR value 35 or less	4	0	4	*Well, the nephrologists have said [to refer for] a GFR for less than 35 …So I do. Period. But I have patients who don’t quite make that….clearly, somebody who has a big jump, without a reason for it, [needs] to have a conversation with the nephrologist, or a referral to the nephrologist. - FP PCP*
				*If in the CKD3 range then just continuing the counseling, lifestyle modifications, and monitoring. And then if it’s dipping into the CKD4 where they’re down in that like 15 to 30 range, I’m thinking renal referral [to Nephrology]. – FP PCP*
· eGFR value 15 to 30	4	0	4	*When they get down to the twenties or especially below, then I usually will refer. Or if they may be Stage 3, but for some reason they seem to be dropping rapidly… Although, usually I’ll work those up first myself. I’ll get an ultrasound and recheck them, and maybe take them off some possible medications and then see what happens. I do all that first, and only if nothing budges …, I usually then ask for a referral. – IM PCP*
· Base it on creatinine not eGFR	0	1	1	*So I don’t refer or ask for chart review from nephrology when eGFR is under 60 though…As far as referral, the threshold is probably closer to 2 on a creatinine. So I use creatinine in that respect. – IM PA*
· did not offer typical cut-off value (based on trends over time)	4	1	5	*I probably don’t have a set number, because it would depend on the whole picture of the patient I want to know if this is a new occurrence or if this is a trend. Is this particular patient a diabetic? If it’s an acute new problem, I would treat it differently than if it’s a chronic ongoing problem. – FP PA*
				*Well, rather than a number, it is a trend… for a patient I’d be looking back and if he or she hadn’t changed much in a year, I wouldn’t be referring.–IM PCP*
***Overall Referral Patterns to Kidney Class***				*I definitely refer more to the class…I like them to go to the kidney class to learn more about how to protect their kidneys. – FP PA*
· refer more to class now	1	1	2	
· refer infrequently to occasionally	4	3	7	*When people are anxious, I will refer to the class for reassurance purposes and education. But I don’t send a high percentage of patients to that class, and I don’t take advantage of that resource as often as I could. – IM PA*
				*I’m aware of it, and I might have referred one person in the past couple of years but usually it’s for the lower GFR’s – like a low 30 – to have them look at their diet and things. But so far I haven’t used that class that much. – FP PCP*
· never refers to class	2	0	2	*I’ve never referred to the kidney class myself. I have the impression that patients who go to kidney class are really the ones who already saw the nephrologist, or if they [Nephrology] advise me in a chart [consult]… - IM PCP*
· no awareness of class/did not mention	6	2	8	*I didn’t even know about it [kidney class]… there you go, I learned something. – IM PCP*

Most clinicians (74%) said that they had increased their overall referrals to Nephrology, but only very slightly. Four of the 13 physicians did not believe eGFR automated reporting had any impact on their referrals, as they still tended to manage and treat their patients up to a later CKD stage of 4 prior to referring. However, all the allied health practitioners reported a perceived increase in referrals, and allied health practitioners were more likely to refer at higher eGFRs (i.e. for less sick patients) than physicians. Physicians divided evenly between referring at late CKD stage 3b and 4, while most allied health reported referring to nephrology at earlier CKD stage 3a. Slightly less than half the clinicians (47%) reported ever referring to the HMO’s ‘kidney class’, a dietician-led class aimed at helping patients take a greater role in their kidney health; most clinicians said their referral pattern to this class did not change with eGFR reporting.

### Suggestions to improve utilization of automated eGFR reporting in clinical practice

The clinicians we interviewed had several suggestions for improving the utilization of automated eGFR reporting, and for improving their overall CKD management. Ongoing clinician education, using a case-study approach, was noted as something they desired and suggested these trainings could be made available both in-person and on-line. They reported being especially interested in 1) why it is better to use eGFR (versus serum creatinine), 2) how eGFR should be used clinically at different CKD stages, and 3) best ways to communicate to patients about their eGFR at different CKD stages (Table 5).

**Table 5:** Suggestions for Future Needs to Improve Utilization of eGFR Value and Overall CKD Management (n = 19)

**Common Theme and Key Findings**	**Illustrative Quotes**
***Ongoing Provider Education***	*It would be nice to get a little lecture on it, and to talk to a nephrologist one-on-one… Or if they could do it, they could make one of those DVD kinds of things and you can tune in or watch at home.* - IM PCP
· yearly trainings both in-person and on-line	
· trainings to focus on: why use eGFR; how to best use it at different states/values; how to best communicate and educate patients at different values/stages	*I’d like to know if the eGFR so sensitive that it’s going to give some false positives, what’s the rate of those that are occurring? And how does our computer monitoring systems handle that… I would like to know more about that*. – NP IM
· provide case-study approach highlighting different patient scenarios	*A good topic to have at one of our CME presentations would be a talk about it in a very case study practical sense; not as a lecture …but more like, okay, let’s look at this person… And just talk us through it, how it’s useful - just the practical use of GFR versus BUN, creatinine in a pure sense – that would be helpful….* – FP PA
· provide both opportunity and responses to provider questions/concerns	
***Regular Feedback from Nephrology to PCPs***	*So there still are questions around what do we miss by just getting a UA and an ultrasound? Is there something more we’re missing, before we send them to the nephrologist and they go on dialysis? What if we’re missing a multiple myeloma or vasculitis, which can be treated before they were sent to the nephrologist. …that’s the type of question I have, ‘Are we getting everything with that UA and the baseline ultrasound?*’ – IM PCP
· Provide yearly to twice yearly feedback on the provider’s actions related to such things as:	
→ referral patterns to Nephrology appropriately – is it too much or coming too late	
	*The Nephrology Department can see the referrals coming in, so they can see how providers, in general, [treat] kidney disease. Are we reasonable with our referrals?…Are we sending people too early or too late? It would be nice to know are there places where there’s room for improvement. I want to know whether I’m doing a reasonable job or not*. – FP PCP
→ ordering patterns for follow up labs and tests - are the appropriate labs and tests being ordered at the appropriate times	
→ identification of whether there is anything else the provider could be doing for the patient both prior to and after referral to Nephrology	*If it’s [eGFR] in the fifties, should I monitor it every 6 months? In the forties, should I monitor it every 4 months? I don’t really know how often I should be monitoring the GFR. And then, when am I supposed to do any other evaluation for their kidneys? …I’m not sure when I’m supposed to do theses other follow up tests*. – FP PA
***Development of Provider Tools/Reminders***	*I had a nice little, laminated handout that came from Nephrology on guidelines and referrals. It has now gone missing, so it would be helpful to have that resent out again – it’s a very convenient and worthwhile thing to have.* - PA IM
· update and re-send out laminated card summarizing current CKD guidelines and “best practice” referral patterns based on eGFR value	
	I guess I would like a dot phrase*. What should I tell these people? What does Nephrology want us to tell people with CKD? …So having a created dot phrase would be wonderful to put on the results to the patient, explaining what to expect and when to come back … - FM NP
· create several different letter templates and phone scripts (based on eGFR value and CKD staging) for use by providers and medical assistants in their discussions and communication with members	
	*It might be really worthwhile to just reorient people, maybe once a year, with an email saying, this is why we’re doing eGFRs, we’ve got physic physician support accompanying that EGFR process, here is what we hope to end up doing with the data, and here’s how we manage those populations of people that are getting chronic kidney disease.* - FM NP
· yearly reminders of where to access CKD guidelines on-line, and any changes in the guidelines	
· yearly reminders of the Kidney class option, including where, when, and how often it occurs and the appropriate circumstances to refer patients to class	*My suggestion would be to offer another quick link in the clinical practice guidelines to take you there to quickly find CKD guideline information*. – FP PCP
	*I’d love to see a promotion about the kidney class, so that clinicians are more aware of it. … if they’d promote the kidney class and say, in general these classes are offered at[these] various times and locations, etc. -. that would help primary care, because we inevitably get those types of questions*. – FP PCP
***Improved Integration of GFR values into EMR/Other Web-based Tools***	*In primary care, we’ve set up this thing called Relevant Results. It can show up either on our scheduled page or when we open the patient’s chart. It gives the trend of three things, but we don’t have the GFR there. We just have the creatinine… it would be nice and helpful to have the GFR there as well*. – FM PCP
· consistent, automatic process for eGFR value and follow ups to be reported in commonly used areas of the EMR – such as patient problem list; results reporting; and trended results	
	*I would like a “decreased GFR” option for the problem list… When I put a problem in the problem list, I just have to pick something that is close to what I want, so for this I’ve chosen the ‘elevated creatinine’ option as a flag, but what I really want is a ‘decreased GFR’ option.* -FM NP
· improve ability of computer to correctly impute race so providers and patients see only one eGFR value rather than both on lab results, outreach prompts, or patient letters	
· continue to improve and refine smart set tools in the internal referral process of EMR to facilitate proper lab orders and follow up by providers	
***Patient-Related Education Tools***	*On the subject of kidney health, I would like some models, pictures, charts, those sorts of things that would be useful in discussion with patient - something that that you could use to pin to your wall and say, ‘Now here’s what a kidney looks like and this is the problem with the low GFR’, for example.* – IM PA
· create standard, uniform hand-outs for providers to use with patients to help explain kidney functioning, meaning of eGFR values, and CKD staging	
· create visual exam room posters of the kidneys and how they function to assist with provider communication and education to patients	*I try to say to patients their kidneys aren’t failing, they’re just not functioning optimally. That part I don’t have worded right, because some of them tend to freak out about it, so I would like some help with wording that, and how to explain that to them…if we could just give them a packet, I think if they would read that, that might be helpful. But the wording, I would like some help with that, because I don’t seem to quite get that right*. - FP PA
· improve patient information and education about CKD and kidney functioning on the organization’s external website	
	*I would love to have a chronic kidney disease patient handout that gives the basic information. It would be good if we had a specialist-created, kind of supervised handout that we can give to patients that says, ‘Here’s what you should do to control your blood pressure, your diabetes if you have it; avoiding these substances that can be harmful to the kidneys. And here’s what you should do for nutrition, protein intake, water intake, etcetera’. And have that kind of be standardized… And that information could then also be easily found by patients online at KP.org, so if they’re looking for CKD information it is there online as well.* – FP PCP

* *dot phrase:* an electronic medical record tool of pre-populated, standardized text that a provider can automatically insert into such things as letters, after visit summaries, and lab results for patient viewing. Dot phrases may be designed individually by providers or by specialty departments such as Nephrology.

Clinicians were also interested in hearing feedback, on an on-going basis, from nephrology on their actions related to CKD care. They particularly mentioned desiring feedback on timing of nephrology referrals, ordering of follow-up laboratory tests and the timing if these tests, and clinical care they should be providing both before and after nephrology referral.

Other specific types of needs identified were related to clinician tools and reminders. In the past, the HMO’s department of nephrology supplied laminated 4″ × 6″ cards that summarized CKD guidelines and best practices. Several clinicians, particularly allied health practitioners, mentioned that they had found these helpful and would like them updated and made available. Clinicians also desired help with patient communication templates for both letters and telephone scripts that could be used by them and their medical assistants. They also mentioned it would be helpful to have reminders of where to access CKD guidelines and reminders of options for patient education such as the aforementioned kidney class.

Clinicians had several suggestions for the integration of the eGFR reporting into the EMR. Some of these suggestions had to do with reminders to obtain follow-up laboratory measures, perhaps incorporated into patients’ diagnosis list, in the laboratory values reports, and in the section of the EMR that reports trended laboratory values. They also discussed the need to address the confusion over the eGFR report containing two values that depend on race. Clinicians also expressed a desire to see improvements in the ability of the EMR to facilitate appropriate laboratory orders and follow-up, for example through ‘smart sets’ that automatically allow a pended order for future laboratory kidney-related tests.

The clinicians we interviewed were also keen to see more patient related education tools, including handouts that explain kidney function, the meaning of eGFR and CKD staging. They said exam room posters of kidney function could facilitate communication with patients, and that the HMO’s external website could be used to improve communication about kidney health.

## Discussion

We found that clinicians were aware of eGFR reporting and generally held favorable views toward it, but also noted some barriers to its use. Perhaps the most interesting theme that emerged from our interviews was that eGFRs were not used to replace serum creatinines, but were used as an added source of information. Clinicians used eGFR as a tool to help: 1) identify CKD; 2) educate patients about their kidney function and; 3) make treatment decisions. The clinicians we interviewed suggested that the added gradation provided by eGFR allowed them to identify CKD at earlier stages than serum creatinine alone, but for most of the clinicians we interviewed the eGFR did not replace serum creatinine as an indication of later staged kidney disease.

It appears from clinician responses that serum creatinine is used as a means of validating eGFR measures. While this may seem redundant, it may be entirely appropriate. Though serum creatinine overestimates renal function when it is poor, eGFR underestimates renal function when it is normal [7,24]. A number of comments by the clinicians we interviewed highlight that concern. Undue stress and fear expressed by patients and the increased burden of tracking patients who may have normal kidney function seem likely to be the result of GFR underestimation. This finding may help confirm prior efforts to measure the cost/effectiveness ratio of eGFR reporting which have found that while eGFR may be beneficial to patients with CKD the benefit was offset by false positive diagnoses of CKD [25], At KPNW we have subsequently switched to the CKD-EPI formula which reduces the effect of underestimation of GFR at normal and near normal levels.

Reporting of eGFR has seemingly created a greater awareness of kidney dysfunction among the clinicians we interviewed. This is a significant finding because that enhanced awareness highlights shortcomings in clinician education; in fact, suggestions made by the clinicians we interviewed to improve utilization of eGFR value revolved primarily around clinician education. Interaction between nephrologists and primary care physicians would appear to play an important role in how eGFRs are utilized. KPNW has made efforts to educate primary care clinicians (CME conferences, written literature, guidelines embedded in the EMR), and the message from our study illustrate that ongoing educational efforts are important. It may also suggest that, because busy primary care clinicians can’t always avail themselves of these opportunities, it is incumbent on the system to advertise the educational opportunities widely, on an on-going basis, and offer several venues to accommodate varied learning and practice styles. The need for clinician education is likely to be greater in other medical systems that have not undertaken similar efforts.

Our study was qualitative, meaning it lacks the empiric information necessary to discern whether the responses represented the feelings of clinicians across the Kaiser Permanente system, or whether they can be extrapolated to other clinicians and medical systems. For example, our findings are specific to a health system with an extant, fully functioning EMR. Such a system may allow clinicians more immediate access to ancillary information (e.g. guidelines) about eGFR interpretation, perhaps easing the transition. The opinions expressed may have been different if the interviews were conducted by a different interviewer or if solicited by another means (i.e. a survey). Additionally, our modest number of interviews may yield less stable frequency estimates than if we had access to a larger sample. But strengths of our approach include the use of a pre-specified interview guide, use of trained interviewers, and interviewing to “saturation”.

## Conclusion

The manner in which clinicians use eGFRs appears to be more complex than previously understood, and our study illustrates some of the efforts that might be usefully undertaken (e.g. specific clinician education) when encouraging further promulgation of eGFR reporting and usage.

## Competing interests

The authors declare they have no competing interests.

## Authors’ contributions

DS, JS, MT, SV, JW, EJ, AF, SS contributed to the methodology, study design, analysis and drafting of the manuscript. AP and XY performed the analysis of the material and drafting of the manuscript. JS also conducted the interviews. All authors read and approved the final maunscript.

## Pre-publication history

The pre-publication history for this paper can be accessed here:

http://www.biomedcentral.com/1471-2369/13/154/prepub
